# Theoretical models of synaptic short term plasticity

**DOI:** 10.3389/fncom.2013.00154

**Published:** 2013-10-30

**Authors:** Matthias H. Hennig

**Affiliations:** School of Informatics, Institute for Adaptive and Neural Computation, University of EdinburghEdinburgh, UK

**Keywords:** short term plasticity, Synaptic Transmission, mathematical model, synaptic depression, synaptic facilitation

There is a misprint in Equation 11, which should read:

(11) $$CV=\sqrt{\frac{1-p(t)}{N(t)p(t)}}$$

